# Revision of the *Lacinipolia vicina* (Grote) complex (Noctuidae, Noctuinae, Eriopygini)

**DOI:** 10.3897/zookeys.527.9686

**Published:** 2015-10-15

**Authors:** B. Christian Schmidt

**Affiliations:** 1 Canadian National Collection of Insects, Arachnids and Nematodes, Agriculture and Agri-Food Canada, K.W. Neatby Bldg., 960 Carling Ave., Ottawa, ON, Canada K1A 0C6

**Keywords:** Cryptic species, Pacific Northwest, California

## Abstract

The *Lacinipolia vicina* (Grote) species complex, previously consisting of *Lacinipolia vicina*, *Lacinipolia teligera* (Morrison), *Lacinipolia pensilis* (Grote), and *Lacinipolia subalba* Mustelin is revised to six species: *Lacinipolia vicina* (eastern USA), *Lacinipolia teligera* (southern Great Plains), *Lacinipolia pensilis* (Pacific Northwest and northern Rocky Mountains), *Lacinipolia acutipennis* (Grote), **stat. rev.** (= *Lacinipolia subalba* **syn. n.)** (western North America), *Lacinipolia sareta* (Smith), **stat. rev.** (Canada and western USA) and *Lacinipolia dimocki*, **sp. n.** (California and Pacific Northwest). Lectotypes are designated for *Lacinipolia vicina*, *Lacinipolia teligera* and *Lacinipolia pensilis*.

## Introduction

*Lacinipolia* McDunnough is currently one of the largest North American noctuid genera with 61 species, and includes another 10 species described from Mexico and Central America. The diversity centers for *Lacinipolia* are the arid habitats of the American Southwest and Mexico. Like many of the constituent genera of the Eriopygini, a diverse, largely New World tribe, the current concept of *Lacinipolia* is not monophyletic and in need of revision. Lloyd Martin initiated this considerable undertaking in the 1960s, but abandoned the project after the loss of all his notes and type photographs (Leuschner 1992). Selman (1975) based his unpublished thesis dissertation on the earlier work of Lloyd Martin, and since Selman’s thesis was never published, Selman and Leuschner (2001) described the nine new species treated therein. Within *Lacinipolia* (*sensu stricto*), the *Lacinipolia vicina* (Grote) group previously consisted of four species, here revised to six species.

## Methods and materials

Adult genitalia were prepared following the methods of Lafontaine (2004). Cleaned, stained genitalia were stored and examined in 30% ethanol, and slide-mounted in Euparal before being photographed using a Nikon D200 digital camera. Distribution maps for examined material were generated using SimpleMappr (Shorthouse 2010).

### Repository abbreviations are as follows

AMNH American Museum of Natural History, New York, NY

BMNH The Natural History Museum (statutorily: British Museum [Natural History]), London.

CNC Canadian National Collection of Insects, Arachnids and Nematodes, Ottawa, ON

USNM National Museum of Natural History (formerly United States National Museum), Washington, DC

MCZ Museum of Comparative Zoology, Cambridge, MA

MSU Michigan State University, East Lansing, MI

CUIC Cornell University, Ithaca, NY

Variation of the ‘barcode’ section of the COI gene was compared among 264 specimens representing all six species (Suppl. material 1). DNA extraction, PCR amplification, and sequencing of the COI barcode region were performed at the Canadian Centre for DNA Barcoding (CCDB) and followed standard protocols (Hajibabaei et al. 2005; Ivanova et al. 2006; deWaard et al. 2008; Hebert et al. 2013; http://www.ccdb.ca/resources.php). PCR and sequencing generally used a single pair of primers: LepF1 (ATTCAACCAATCATAAAG ATATTGG) and LepR1 (TAAACTTCTGGATGTCCAAAAAATCA) (Hebert et al. 2004) which recovers a 658 bp region near the 5' end of COI including the 648 bp barcode region for the animal kingdom (Hebert et al. 2003). Only sequence records greater than 500 bp (range 500 bp–658 bp) are included.

## Systematics

### Key to species of the *Lacinipolia vicina* group

**Table:** 

1	Male	**2**
–	Female	**7**
2	Spines posterior to juxta (‘above’ juxta in slide preparations) pointing antero-ventrally and forming spinose crests or simple patch^1^ (Figs 55–58)	**3**
–	Spines posterior to juxta pointing postero-dorsally and situated on inside surface of spade-like plate (Figs 59, 60)	**6**
3	Medial field of ventrally projecting spines located adjacent to juxta (Figs 55, 56)	**4**
–	No medial spine patch adjacent to juxta(Figs 57, 58)	**5**
4	Clasper with thumb positioned at basal third of distance to apex (Fig. 55); hindwing with dark fuscous terminal shade (Figs 1, 2); occurring east of the Mississippi Valley (Fig. 69)	***Lacinipolia vicina***
–	Clasper with thumb positioned nearly halfway to apex (Fig. 56); hindwing without dark fuscous terminal shade (Figs 4, 5); occurring west of the Mississippi Valley (Fig. 70)	***Lacinipolia teligera***
5	Crest of phallus usually with a thin, delicate apically-directed spine (sometimes broken off, in which case base is still evident) (Fig. 61), or if thin spine absent, then entire crest reduced with fewer and smaller cornuti (Fig. 61c); forewing ground colour highly variable, but medial area concolourous with postmedial and antemedial areas, and antemedial line absent or poorly defined; usually with apical pale area that extends through postmedial line into reniform spot; subterminal area usually darker than postmedial area; reniform and orbicular spot often only faintly visible; orbicular spot sometimes flattened and elongated; arid low elevation habitats including shortgrass prairie and sagebrush steppe	***Lacinipolia acutipennis***
–	Crest of phallus never with a thin, delicate basally-directed spine (Fig. 62), rarely with robust cornutus directed apically (Fig. 62h); forewing ground colour varying in saturation but consistent in tone, with medial area containing brown tones that are lacking in the grey-and-black postmedial and antemedial areas; antemedial line usually well defined; pale apical area not extended through postmedial line; subterminal area not darker than postmedial area; reniform and orbicular spot conspicuous, paler than ground; orbicular spot never highly flattened and elongated; low to high elevation woodland, particularly dry, montane pine and Douglas-fir woodlands	***Lacinipolia pensilis***
6	Clasper with a thumb-like process on ventral margin, clasper flattened and apex rounded; digitus pointed (Fig. 59); widely distributed, including West Coast states (Fig. 73)	***Lacinipolia sareta***
–	Clasper without process, shaped like a sinuate spine with a pointed apex; digitus rounded (Fig. 60); West Coast states (Fig. 74)	***Lacinipolia dimocki***
7	Ostium asymmetrical, like opening of a conch; margin of prevaginal plate straight or slightly convex (Figs 63–66)	**8**
–	Ostium symmetrical, opening simple; margin of prevaginal plate strongly convex (Figs 67, 68)	**1**
8	Ostium complex 1.4–1.5 × longer than wide; caudal portion of ostial slit gradually curved (Figs 65, 66)	**9**
–	Ostium complex 1.0–1.1 × longer than wide; caudal portion of ostial slit sinuate (Figs 63, 64)	**10**
9	Forewing ground colour highly variable, but medial area concolourous with postmedial and antemedial areas, and antemedial line absent or poorly defined; usually with apical pale area extended through postmedial line into reniform spot; subterminal area usually darker than postmedial area; reniform and orbicular spots often only faintly visible; orbicular spot sometimes flattened and elongated; arid low elevation habitats including shortgrass prairie and sagebrush steppe	***Lacinipolia acutipennis***
–	Forewing ground colour varying in saturation but consistent in tone, with medial area containing brown tones that are lacking in grey-and-black postmedial and antemedial areas; antemedial line usually well defined; pale apical area not extended through postmedial line; subterminal area not darker than postmedial area; reniform and orbicular spot conspicuous and paler than ground; orbicular spot never highly flattened and elongated; low to high elevation woodlands, particularly dry, montane pine and Douglas-fir woodland	***Lacinipolia pensilis***
10	Basal half of hindwing conspicuously lighter than marginal portion and forewing (Fig. 6); occurring in southern Great Plains west of Mississippi River (Fig. 70)	***Lacinipolia teligera***
–	Basal half of hindwing nearly as dark as marginal portion and forewing (Fig. 3); occurring in the eastern United States east of Mississippi River (Fig. 69)	***Lacinipolia vicina***
11	Ductus bursae highly flattened dorsoventrally, with pronounced ribbon-like oblique fold (Fig. 67); corpus bursae 2–2.5 × diameter of ducts bursae; widely distributed, including West Coast states (Fig. 73)	***Lacinipolia sareta***
–	Ductus bursae moderately flattened dorsoventrally, with slight oblique fold; corpus bursae 3–4 × diameter of ductus bursae (Fig. 68); West Coast states from Washington to California (Fig. 74)	***Lacinipolia dimocki***

**Figures 1–27.**
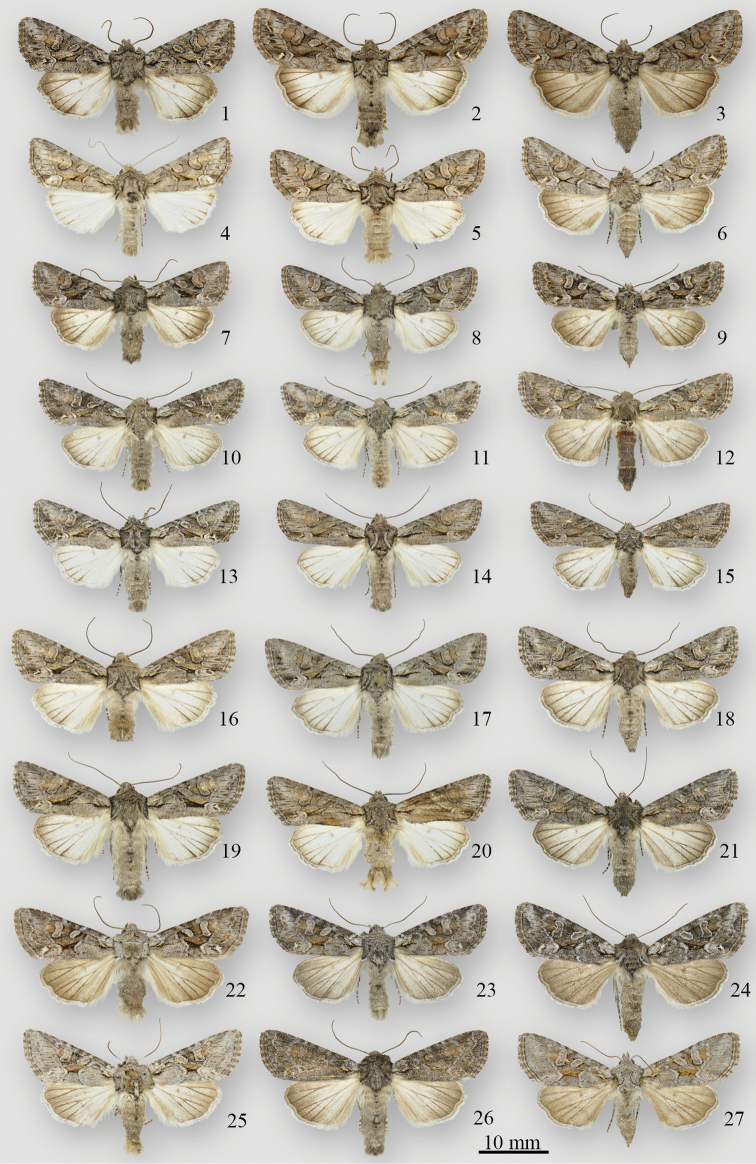
*Lacinipolia* adults. **1** *Lacinipolia vicina* ♂ NC, Watauga Co., Beech Ck. bog **2** *Lacinipolia vicina* ♂ PA, Beaver Co., 6 mi SW Darlington **3** *Lacinipolia vicina* ♀ PA, Beaver Co., 6 mi SW Darlington **4** *Lacinipolia teligera* ♂ TX, Lampasas Co., Lampasas R. 8 mi S Rte. 190 **5** *Lacinipolia teligera* ♂ TX, Travis Co., Austin **6** *Lacinipolia teligera* ♀ TX, Lampasas Co., Lampasas R. 8 mi S Rte. 190 **7** *Lacinipolia sareta* ♂ ON, Manitoulin Is., Dominion Bay dunes **8** *Lacinipolia sareta* ♂ BC, [Lillooet], Kirby Flats Rd. **9** *Lacinipolia sareta* ♀ ON, Manitoulin Is., Sheguindah **10** *Lacinipolia sareta* ♂ AB, Waterton Lakes NP, Blakiston Ck. fan **11** *Lacinipolia sareta* ♂ AB, Manyberries, Pakowki Dunes **12** *Lacinipolia sareta* ♀ AB, Manyberries, Pakowki Dunes **13** *Lacinipolia sareta* ♂ CA, Mono Co., Lee Vining **14** *Lacinipolia sareta* ♂AZ, Cochise Co., Huachuca Mtns, Ash Cyn. Rd. **15** *Lacinipolia sareta* ♀AZ, [Maricopa Co.], Congress **16** *Lacinipolia dimocki* ♂CA, Plumas Co., Jackson Ck., DNA voucher # CNCNoctuoidea7972 **17** *Lacinipolia dimocki* ♂ Holotype, CA, Ventura Co., Cuyama Valley, Apache Cyn. **18** *Lacinipolia dimocki* ♀Paratype, CA, Ventura Co., Cuyama Valley, Apache Cyn. **19** *Lacinipolia dimocki* ♂ WA, Klickitat Co., Simcoe Butte **20** *Lacinipolia dimocki* ♂ WA, Klickitat Co., Munson Prairie **21** *Lacinipolia dimocki* ♀ WA, Yakima Co., South Fork Ahtanum Cr. **22** *Lacinipolia pensilis* ♂ BC, Squamish, Diamond Head Trail **23** *Lacinipolia pensilis* ♂ BC, [11 km WSW Invermere], Watch Peak **24** *Lacinipolia pensilis* ♀ BC, [11 km WSW Invermere], Watch Peak **25** *Lacinipolia pensilis* ♂ UT, [Utah Co.,] 12 mi N Provo **26** *Lacinipolia pensilis* ♂ WA, Yakima Co., Bethel Ridge **27** *Lacinipolia pensilis* ♀ UT, Salt Lake City.

**Figures 28–54.**
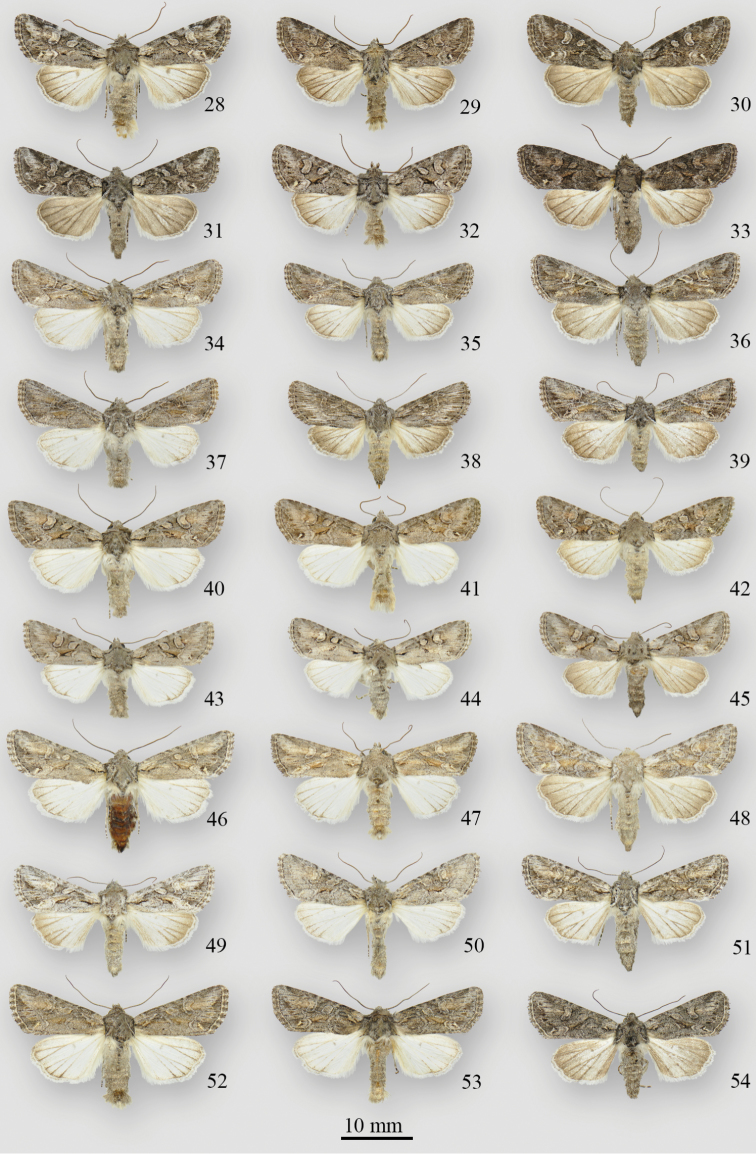
*Lacinipolia acutipennis* adults. **28** ♂ BC, [Lillooet], Kirby Flats Rd. ♂ BC, Savona, 2 mi SW **30** ♀ BC, [Lillooet], Kirby Flats Rd., DNA voucher # CNCNoctuoidea7978 **31** ♀ BC, [Lillooet], Kirby Flats Rd. **32** ♂ WA, [Okanogan Co.], Tonasket, 8 mi S **33** ♀ CA, Plumas Co., Jackson Ck. **34** ♂ WA, Douglas Co., Jameson L. **35** ♂ WA, Grant Co., Dodson Rd. **36** ♀ WA, Yakima Co., South Fork Ahtanum Cr. **37** ♂OR, Crook Co., Suplee **38** ♀ OR, Lake Co., Alkali L. **39** ♀ OR, Baker Co., Burnt River, 20 mi S **40** ♂ CA, Ventura Co., Cuyama Valley, Apache Cyn. **41** ♂ CA, [Monterey Co.], Pinnacles Nat. Mon. **42** ♀ CA, [Monterey Co.], Pinnacles Nat. Mon. **43** ♂ [*subalba* paratype] CA, San Diego Co., S rim Peñasquitos Cyn. **44** ♂ [*subalba* paratype] CA, San Diego Co., S rim Peñasquitos Cyn. **45** ♀ [*subalba* paratype] CA, San Diego Co., NAS Miramar 6 **46** ♂ AB, Dinosaur PP **47** ♂ MT, [Philips Co.], Malta, 19 mi NE **48** ♀ WY, [Albany Co.], Laramie **49** ♂ UT, [Juab Co.], Eureka **50** ♂ ID, Owyhee Co., [Castle Ck. Rd.] **51** ♀ UT, Grand Co., Thompson Cyn. **52** ♂ CO, Adams Co., Bennett **53** ♂ CO, Larimer Co., Flatiron Reservoir **54** ♀ CO, Larimer Co., Flatiron Reservoir.

**Figures 55–57.**
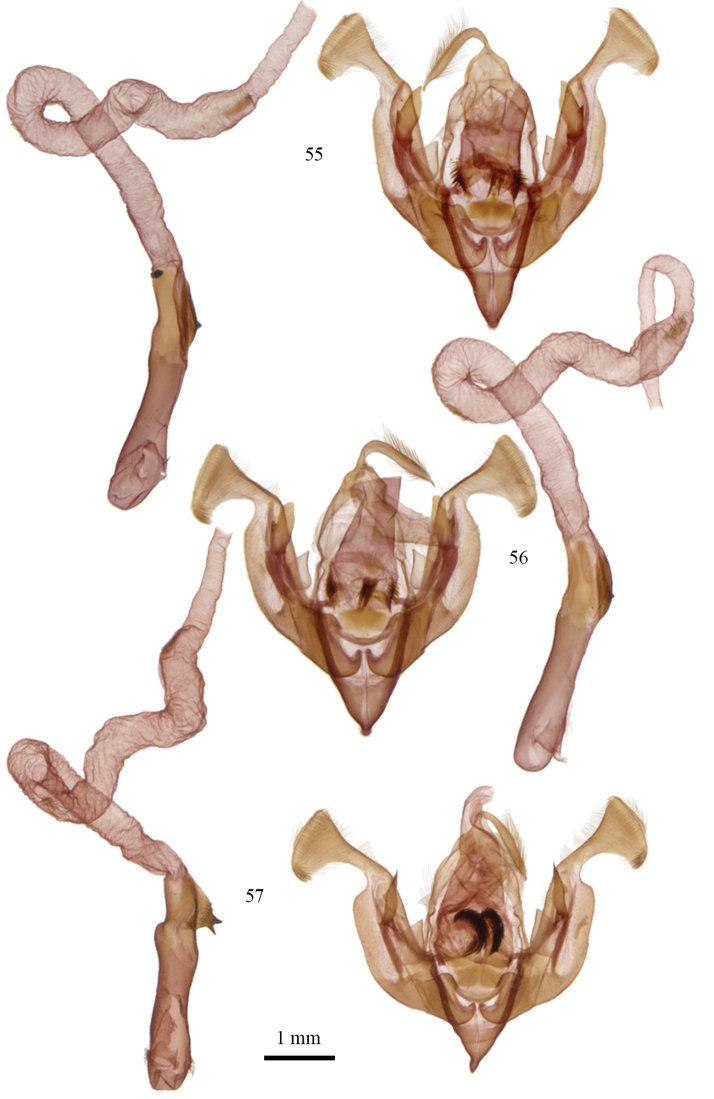
*Lacinipolia* male genitalia. **55** *Lacinipolia vicina* MA, Barnstable, CNC Gen. Prep. # CNCLEP16884 **56** *Lacinipolia teligera* TX, 6 mi E Canadian, CNC Gen. Prep. # CNCLEP16886 **57** *Lacinipolia pensilis* BC, Crowsnest, 5 mi NW, CNC Gen. Prep. # CNCLEP16852.

**Figures 58–60.**
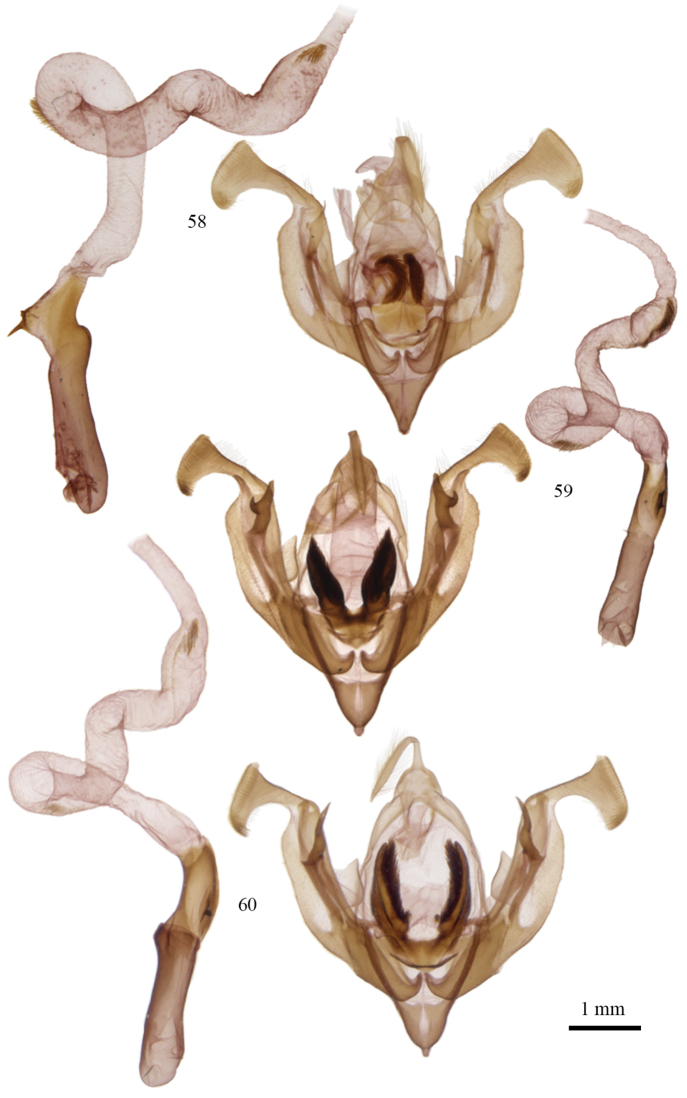
*Lacinipolia* male genitalia. **58** *Lacinipolia acutipennis* AB, Steveville, CNC Gen. Prep. # CNCLEP16843 **59** *Lacinipolia sareta* AZ, Prescott, CNC Gen. Prep. # CNCLEP16867 **60** *Lacinipolia dimocki* CA, Mt. Laguna, CNC Gen. Prep. # CNCLEP16871.

**Figure 61.**
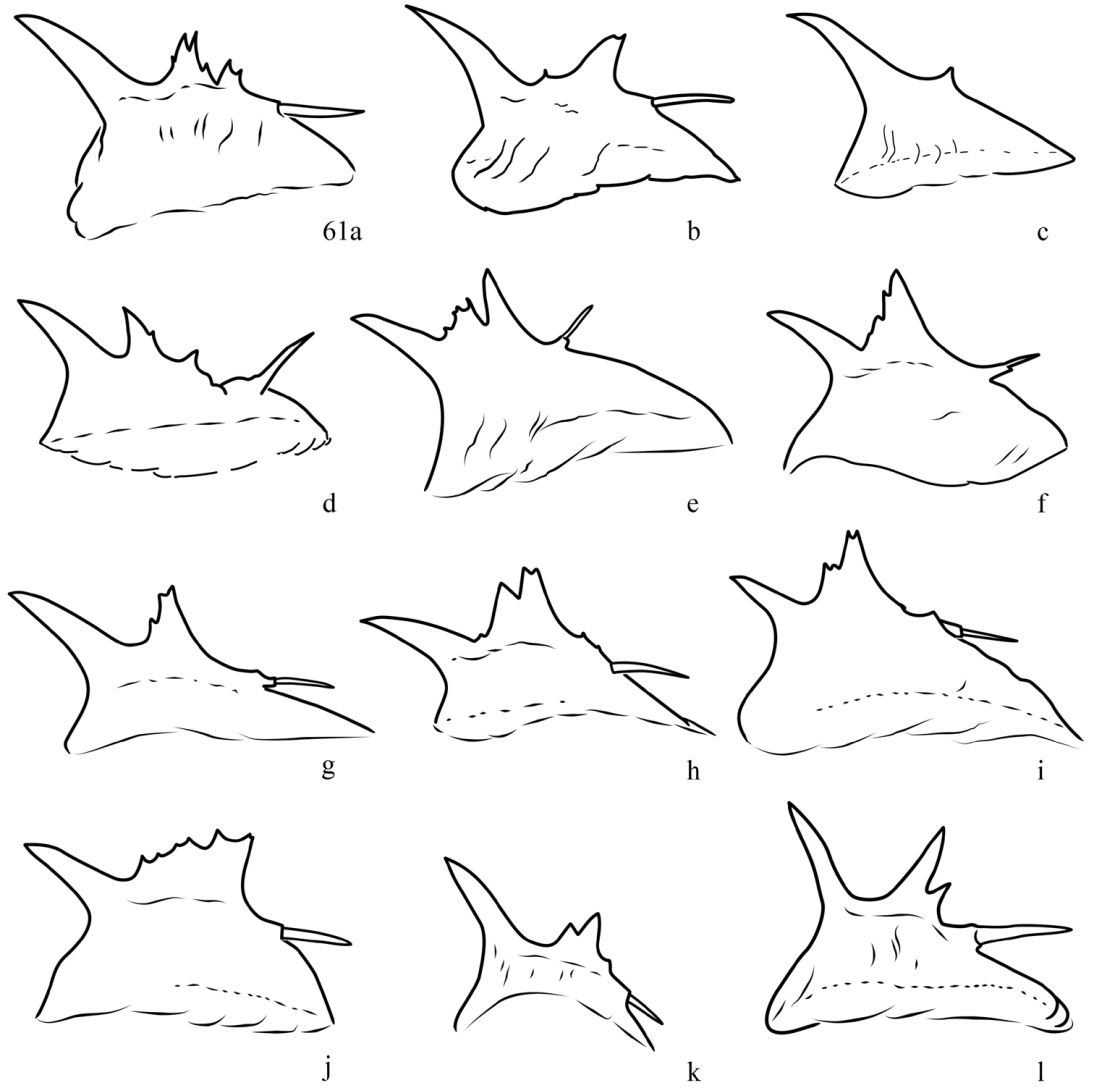
Variation in spined crest of male phallus, *Lacinipolia acutipennis*. **a–c** UT, Stockton **d** NV, Ely **e** WA, Omak **f** WA, Tonasket **g–h** OR, Biggs **i** BC, Kamloops **j** BC, Keremeos **k** AB, Dinosaur PP **l** MT, Joliet.

**Figure 62.**
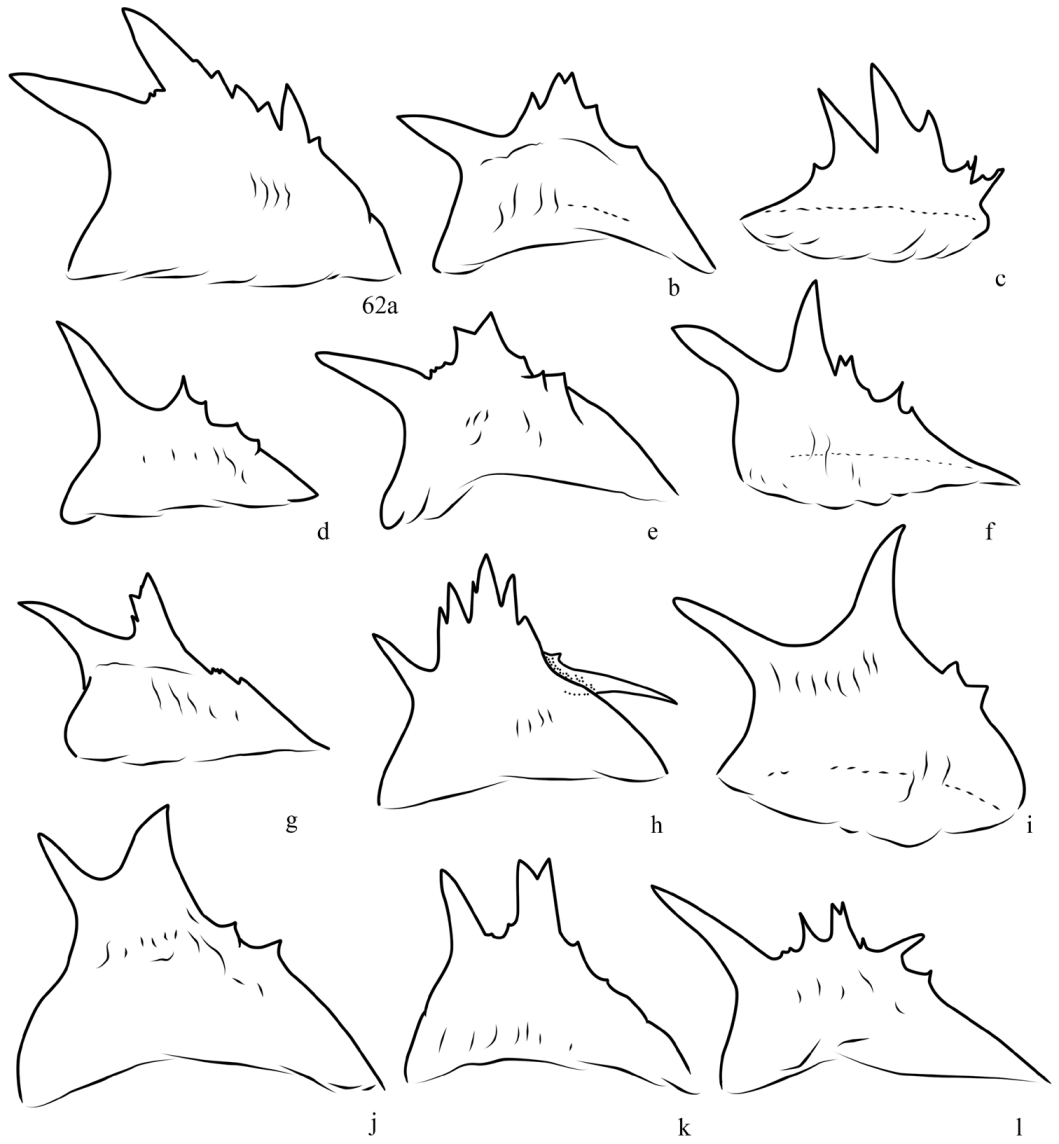
Variation in spined crest of male phallus, *Lacinipolia pensilis*. **a** WA, Satus Ck. **b** UT, Grantsville **c** MT, Brooks **d** UT, Logan **e–f** BC, Squamish **g–h** BC, Wellington **i** BC, Riondel **j** BC, Crowsnest **k** BC, Okanagan Falls **l** BC, Squamish.

**Figures 63–68.**
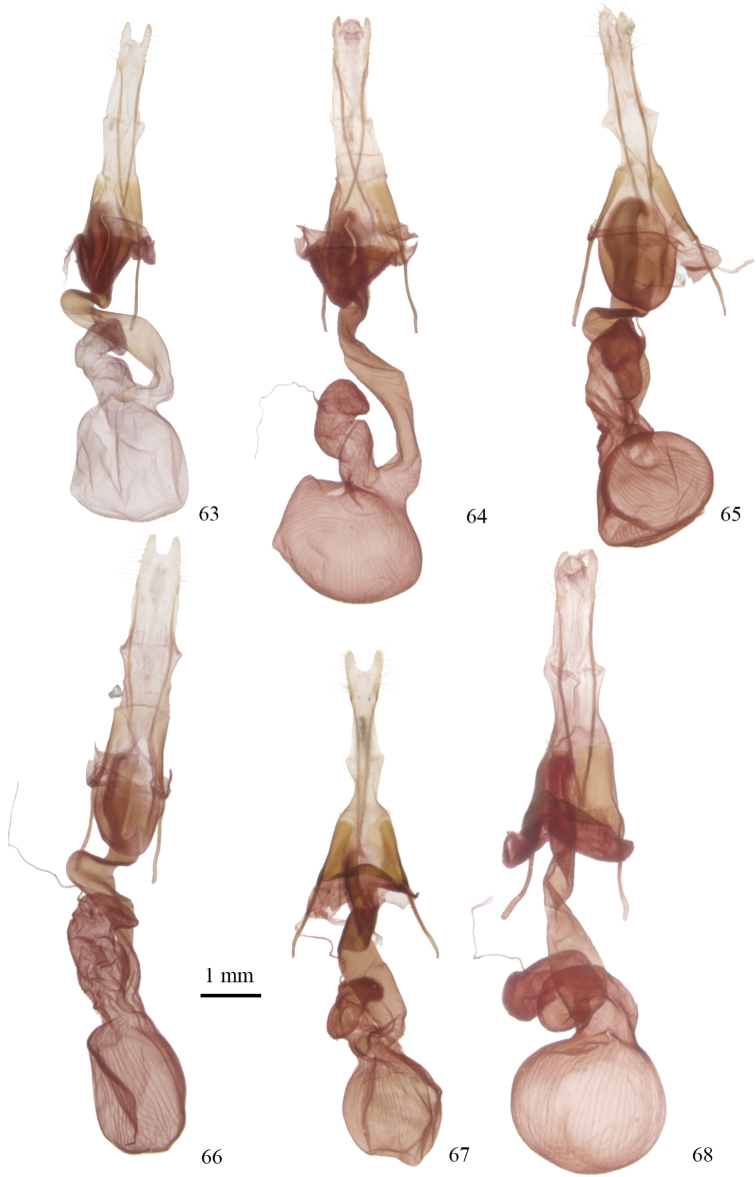
*Lacinipolia* female genitalia. **63** *Lacinipolia vicina* MA, Barnstable, CNC Gen. Prep. # CNCLEP16883 **64** *Lacinipolia teligera* TX, 16 mi ESE Canyon, CNC Gen. Prep. # CNCLEP16885 **65** *Lacinipolia pensilis* BC, Mt. Kobau, CNC Gen. Prep. # CNCLEP16840 **66** *Lacinipolia acutipennis* CA, Truckee, CNC Gen. Prep. # CNCLEP16847 **67** *Lacinipolia sareta* AB, Wainwright Dunes, CNC Gen. Prep. # CNCLEP16837 **68** *Lacinipolia dimocki* CA, Plumas Co., Happy Valley, CNC Gen. Prep. # CNCLEP16881.

**Figures 69–74.**
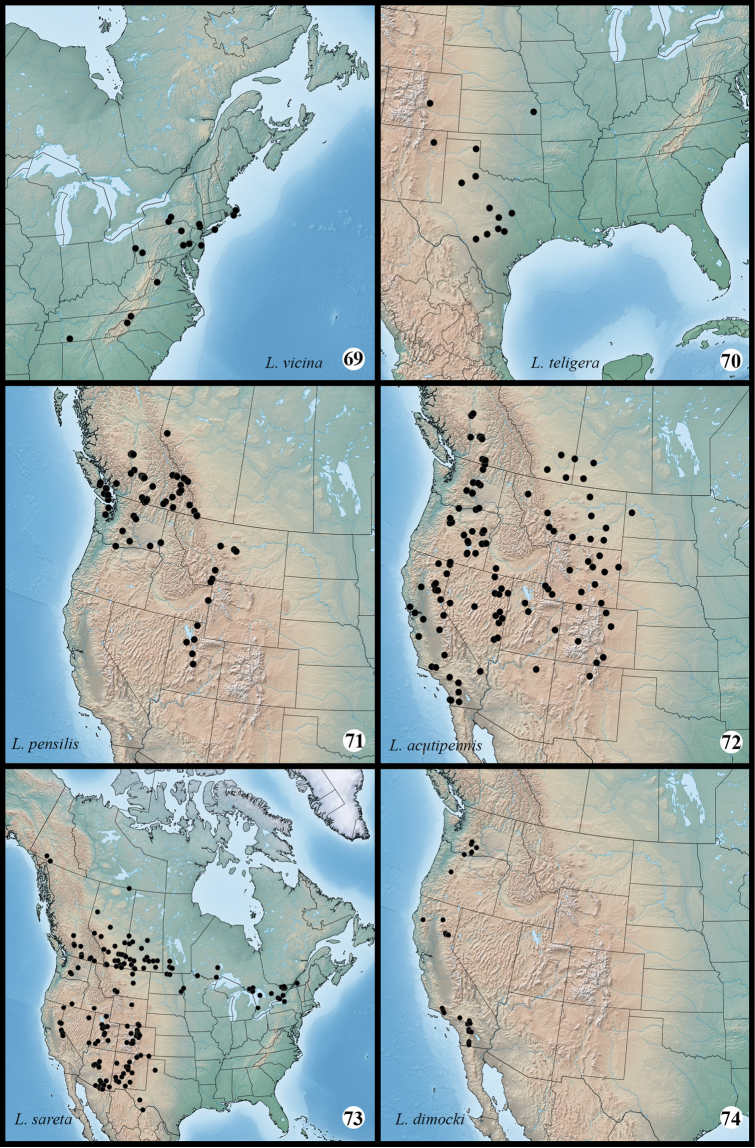
Distribution of examined specimens of *Lacinipolia*.

### Lacinipolia vicina

Taxon classificationAnimaliaLepidopteraNoctuidae

(Grote, 1874)

Figs 1–3 , 55 , 69

Mamestra vicina Grote, 1874a: 156.
Mamestra imbuna Smith, 1905a: 201, **syn. rev.**

#### Type material.

*Mamestra vicina*: The type material of *Lacinipolia vicina* almost certainly consisted of two species, the eastern species known previously as *Lacinipolia imbuna* or *Lacinipolia teligera* (Franclemont and Todd 1983) and represented by a female syntype from Massachusetts (BMNH; examined), in addition to the widespread species previously called *Lacinipolia vicina*, represented by at least one syntype from St. Catherines, Ontario (lost). I was unable to locate any St. Catherines specimens, stated by Grote to have come from George Norman. Other syntypes from the Norman collection (*Crocigrapha normani* (Grote) and *Xestia normanianus* (Grote)) are also considered to be lost (D. Lafontaine pers. comm.). This is unfortunate since it would have been preferable to fix the name *vicina* as the widespread, well-known species here treated as *Lacinipolia sareta*, but as the only extant primary type, the following female specimen [BMNH] must be designated as **lectotype**: “Mamestra / vicina / Type Grote” [red-bordered label]; “Type” [round red-bordered label]; “vicina / TYPE” [small handwritten label]; Grote Coll. / 81-116.” [type-written label]; “U.S.America.” [type-written label]; “Noctuidae / Brit. Mus. slide / No. 8237” [blue type-written label]. Type locality: “Massachusetts”.

*Mamestra imbuna*: Male lectotype (AMNH; examined), designated by Todd (1982). Type locality: Luzerne Co., Pennsylvania. The original type series of *Mamestra imbuna* probably also included *Lacinipolia sareta* from the southern Lake Michigan region, as Smith (1905a) mentions an August specimen from Hessville, Indiana (a suburb of Chicago), but Todd’s lectotype designation fortunately restricts the concept of the name.

#### Diagnosis.

Within the eastern North American range of *Lacinipolia vicina*, *Lacinipolia sareta* is most similar but the two can usually be separated without dissection by the more southern distribution, larger size and bivoltine spring / fall flight (April-May and September - October) of *Lacinipolia vicina* (univoltine from late June to early August for *Lacinipolia sareta*). In the male genitalia, *Lacinipolia vicina* differs most obviously in the arrangement of the spines above the juxta, consisting of two lateral and one medial field of ventrally projecting spines, whereas in *Lacinipolia sareta* the spines are directed dorsally and are on the inside of a large, rhomboid plate. Females of *Lacinipolia vicina* have an asymmetrical, invaginated ostium, like the opening of a conch, compared to a simpler ostium with a convex prevaginal plate margin in *Lacinipolia sareta*.

Although *Lacinipolia vicina* is most closely related to *Lacinipolia teligera*, *Lacinipolia vicina* and *Lacinipolia teligera* are not likely to be confused given the range disjunction and more extensive dark fuscous shading of the hindwing in *Lacinipolia vicina*. The male genitalia differ in the shape of the clasper, with the apical lobe narrower and more pointed in *Lacinipolia vicina*, and the thumb-like lobe situated one third the distance from the base, compared to halfway in *Lacinipolia teligera*.

#### Distribution and biology.

Specimens of *Lacinipolia vicina* were examined from Massachusetts, New York, Pennsylvania, Virginia and North Carolina (Fig. 69); Forbes (1954) also cites New Jersey (Lakehurst) and Indiana records. The Indiana record (Smith 1905a) may be erroneous given the long-standing confusion with *Lacinipolia sareta*, as discussed in the “Type material” section above. Eastern Ohio records of *Lacinipolia teligera* from May and September given by Rings et al. (1992) are most likely *Lacinipolia vicina*. Moore’s (1955) records for Michigan probably all apply to *Lacinipolia sareta* based on flight dates and the widespread distribution of *Lacinipolia sareta* in the Great Lakes region. There is no clear indication of habitat preference; in North Carolina *Lacinipolia vicina* occurs in open oak-hickory forest (B. Sullivan pers. comm.). Despite the relatively broad distribution and apparent lack of specialized habitat requirements, *Lacinipolia vicina* records are few. *Lacinipolia vicina* is apparently bivoltine, flying in spring (April–May) and in late summer to early fall (late August to early October), with later dates farther south. The larvae were described and illustrated by Godfrey (1972) (reared vouchers examined; CUIC), and are probably polyphagous ground dwellers like other *Lacinipolia* (Wagner et al. 2011).

#### Remarks.

As defined here, *Lacinipolia vicina* is the same species later described by Smith (1905a) as *Mamestra imbuna*, differing considerably in morphology from both *Lacinipolia sareta* (= *vicina* of authors) and *Lacinipolia pensilis*, although more closely related to the latter. *Lacinipolia imbuna* was previously treated as a junior synonym of *Lacinipolia teligera* (Franclemont and Todd 1983).

### Lacinipolia teligera

Taxon classificationAnimaliaLepidopteraNoctuidae

(Morrison, 1875)

Figs 4–6 , 56 , 64 , 70

Mamestra teligera Morrison, 1875: 215.

#### Type material.

Morrison’s original description was based on two specimens, and Wilterding (1997) discussed two Morrison specimens in the MSU collection: a male with two conflicting locality labels (Texas and New York), and a damaged female specimen labelled simply “19/10” and probably not a syntype. A third specimen in MCZ (photo available at http://insects.oeb.harvard.edu/mcz/Species_record.php?id=1585), dissected and labelled as follows, is here designated as **lectotype**: “Dianthoecia / teligera / Type / Morr”; “Tex.”; 27/10”; “Certainly not / vicina as / refined by Grote”; “Peab. Acad.”; “Type / 1742”; “M.C.Z. Type # / gen. 1742 / 28 Jan. 33 H[?]. B.”; the following label will be added: “Lectotype / Dianthoecia / teligera Morr., 1875 / desig. by Schmidt 2015”. Type locality: Waco, Texas.

#### Diagnosis.

Although closely related to *Lacinipolia vicina*, the challenge in identifying *Lacinipolia teligera* is in separating it from *Lacinipolia sareta* in the southwestern Great Plains where the ranges of the two can overlap. Compared to *Lacinipolia sareta*, *Lacinipolia teligera* is slightly larger with a broader forewing and better-defined, crisper forewing maculation. Reliable identification should be based on genitalic structure, where *Lacinipolia teligera* males have a medial and lateral field of short, ventrally directed spines above the juxta, rather than two large flanges laterally on the juxta with dorsally directed spines in *Lacinipolia sareta*. The female *Lacinipolia teligera* has an asymmetrical, invaginated ostium (like the opening of a conch), whereas that of *Lacinipolia sareta* has a simple ostium with the margin of the prevaginal plate convex.

#### Distribution and biology.

*Lacinipolia teligera* is known from the Great Plains of central Colorado and eastern Kansas southward to central Texas (Fig. 70). Nothing is known of the early stages, although these are undoubtedly similar to those of *Lacinipolia vicina*.

#### Remarks.

*Lacinipolia teligera* is closely related to *Lacinipolia vicina*, and the two have previously been considered conspecific (as *Lacinipolia imbuna*; Franclemont and Todd 1983). However the two differ structurally as outlined in the *Lacinipolia vicina* diagnosis, in addition to a DNA barcode difference of 1.0%. The two species occupy separate ecoregions and different habitats, with *teligera* in the grasslands of the Great Plains and *vicina* in deciduous forest of the Appalachian and Atlantic region.

### Lacinipolia pensilis

Taxon classificationAnimaliaLepidopteraNoctuidae

(Grote, 1874)

Figs 22–27 , 57 , 61 , 65 , 71

Dianthoecia pensilis Grote, 1874b: 199.

#### Type material.

described from at least 1 male and 1 female syntype; the following male [BMNH] is here designated as **lectotype**: “Dianthoecia / pensilis ♂ / Type Grote” [red-bordered handwritten label]; “Type” [round, red-bordered, typed label]; Vancouver I / Grote Coll. / 81 – 116”; “5597”; Noctuidae / Brit. Mus. slide / No. 4912 ♂” [blue type-written label]; the following label will be added: “Lectotype / Dianthoecia / pensilis Grote, 1874 / desig. by Schmidt 2015.” Type locality: Victoria, [Vancouver Island, British Columbia, Canada].

#### Diagnosis.

*Lacinipolia pensilis* is a northwestern montane species that is often confused with *Lacinipolia sareta* and also *Lacinipolia acutipennis* in parts of the range. Compared to *Lacinipolia sareta*, *Lacinipolia pensilis* flies later (August to September versus June to early August), and differs considerably in genitalic structure of both males and females as outlined in the key and the *Lacinipolia sareta* account.

Separating *pensilis* from dark forms of *Lacinipolia acutipennis*, which are prevalent in montane habitats of the Pacific Northwest, poses the greatest identification challenge in the *Lacinipolia vicina* group. *Lacinipolia pensilis* usually has better-defined forewing markings, richer brown tones in the forewing medial area, and no tendency for streaky pale patches in the forewing apical area; *Lacinipolia pensilis* also averages slightly larger with a broader forewing. The spined crest of the male phallus is more robust and usually with more spines, and never has the thin apically-projecting spine that is normally found in *Lacinipolia acutipennis*. In montane parts of the Pacific Northwest (interior British Columbia, northern and central Washington) habitat can help to separate the two, with *Lacinipolia pensilis* occurring from dry montane woodland to high elevation subalpine forest, whereas *Lacinipolia acutipennis* is characteristic of the dry, low-elevation habitats of the major intermontane valleys. See also remarks in the *Lacinipolia acutipennis* account.

#### Distribution and biology.

This species occurs in the western cordilleran region from central British Columbia and western Alberta southward to at least Washington and central Utah. The distribution pattern suggests it may occur farther south along the Cascade–Coast Ranges through Oregon, and further work is needed to establish the southwestern range limits. The larval description and host plants require clarification since the information given by Crumb (1956) and Godfrey (1972) was probably based on both *Lacinipolia acutipennis* and *Lacinipolia pensilis*. The larvae likely are ground-dwelling, general feeders on shrubs and herbs.

### Lacinipolia acutipennis

Taxon classificationAnimaliaLepidopteraNoctuidae

(Grote, 1880) stat. rev.

Figs 28–54 , 58 , 62 , 66 , 72

Mamestra acutipennis Grote, 1880: 214.
Mamestra doira Strecker, 1898: 7, **syn. rev.**
Mamestra ascula Smith, 1905b: 257, **syn. rev.**
†Polia pensilis ab. indistincta Strand, 1917: 28, **unavailable**; infrasubspecific name.
Lacinipolia subalba Mustelin, 2000: 13, **syn. n.**

#### Type material.

*Mamestra acutipennis*: type female (BMNH; examined); type locality: Nevada. *Mamestra doira*: type female (FMNH, examined); type locality: Utah. *Mamestra ascula*: lectotype male designated by Poole (1982), (AMNH, examined); type locality: Stockton, Utah. *Lacinipolia subalba*: South rim of Los Peñasquitos Canyon, 76 m, San Diego Co., California (SDNHM).

#### Diagnosis.

**Figure 75.**
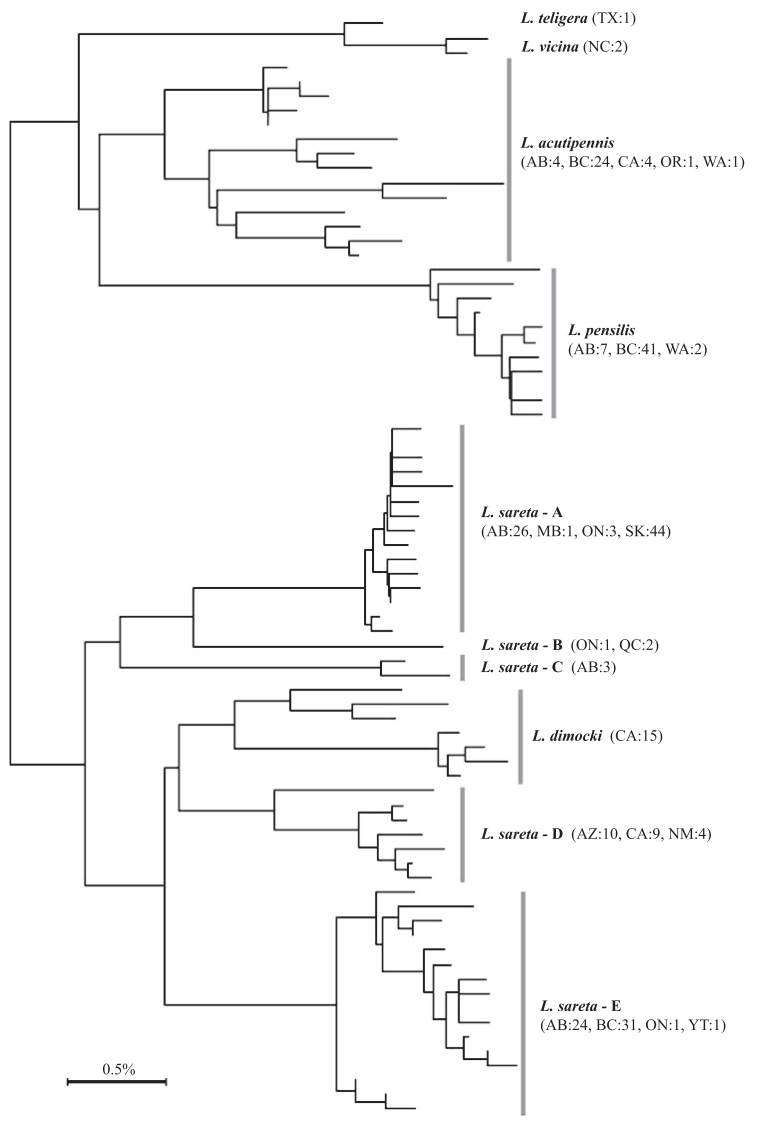
Neighbour-joining tree of representative mtDNA barcode haplotypes in species of the *Lacinipolia vicina* group. Sample size and locality are given in brackets, with number of specimens indicated after two-letter state/province abbreviation. *Lacinipolia sareta* variation is divided into five haplogroups, **A–E**. Voucher specimen data is given in Suppl. material 1.

*Lacinipolia acutipennis* is a western steppe / grassland species that shows considerably greater regional phenotypic variation than others in the *Lacinipolia vicina* group. In more mesic habitats (including higher elevations) of the Pacific Northwest and central Rocky Mountains *Lacinipolia acutipennis* is replaced by the very similar *Lacinipolia pensilis*. The two occur sympatrically in many transitional habitats, mostly dry montane woodlands at moderate elevations. Although phenotypes of *Lacinipolia acutipennis* from the most arid habitats (e.g., Figs 34–39) can be distinguished from *Lacinipolia pensilis* with relative ease, many northern *Lacinipolia acutipennis* populations in the Pacific Northwest are dark, well-marked and very similar to *Lacinipolia pensilis*, which makes identifying the two very difficult and led previous workers to conclude that they represent the same species. Compounding this difficulty is the lack of conspicuous genitalic differences that are otherwise typical of the genus. Despite the identification difficulties in the Pacific Northwest, other sympatric populations of *Lacinipolia acutipennis* and *Lacinipolia pensilis* have clearly different phenotypes. Differences are most pronounced in Great Basin populations (*Lacinipolia pensilis*, Figs 25, 27 and *Lacinipolia acutipennis*, Figs 49–51) and in the northern Rockies/Great Plains (e.g., Montana *Lacinipolia pensilis*, like those in Figs 23, 24, and *Lacinipolia acutipennis*, Figs 46–48). The two differ in male genitalia structure as discussed below. These differences, in addition to a minimum 2.5% divergence in DNA barcodes (Fig. 75), show that (at least) two species are involved.

Similar phenotypes of *Lacinipolia acutipennis* and *Lacinipolia pensilis* differ in the shape and size of the forewing, which averages more acute and smaller in *Lacinipolia acutipennis*; the brown tones of the medial forewing are more muted in *Lacinipolia acutipennis* compared to *Lacinipolia pensilis*, giving an overall lower contrast in tone of the medial area with the grey-black antemedial and postmedial areas; the white spot in the anal angle is often more prominent in *Lacinipolia acutipennis*, particularly in females; the forewing apex has a more contrastingly pale diffuse area that usually extends farther towards the reniform. In the male genitalia of *Lacinipolia acutipennis*, the spinose crest of the phallus usually has a thin, delicate apically-directed spine (which is sometimes broken off, in which case the spine base is still evident), which is absent in *Lacinipolia pensilis*; this thin spine is sometimes absent also in *Lacinipolia acutipennis*, but in such individuals the entire crest is small and with fewer, smaller cornuti (Fig. 61c) compared to *Lacinipolia pensilis* (Fig. 62).

Two phenotypes have been recognized as separate species, *Lacinipolia doira* of the Great Basin (Figs 49–51) and *Lacinipolia subalba* of southern California (Figs 43–45). Clinal phenotypic variation, lack of diagnostic structural characters, and similarity in DNA barcodes, lead me to treat –*doira* and –*subalba* as regional forms.

#### Distribution and biology.

*Lacinipolia acutipennis* is a western species common throughout xeric, low elevation habitats of western North America. The core range includes the dry, western portions of the Great Plains, the Great Basin, and the western intermontane valleys north of the Sonoran zone, from southern Saskatchewan and Alberta southward to northern Arizona and New Mexico. Reports from Wisconsin (cited in Forbes 1954), Texas and southern Arizona (Hampson 1905) are probably misidentifications of *Lacinipolia sareta*. Crumb’s (1954) records from Nebraska and Kansas are plausible; the easternmost specimens I examined were from Watford City in western North Dakota. In the intermontane valleys west of the Rocky Mountains *Lacinipolia acutipennis* occurs from southern British Columbia to southern California and northernmost Arizona and New Mexico (Fig. 72). All Pacific Northwest specimens examined from subalpine habitats and from sites west of the Coast Ranges proved to be *Lacinipolia pensilis*.

The larval description and host plants require clarification since the information given by Crumb (1956) and Godfrey (1972) was probably based on both *Lacinipolia acutipennis* and *Lacinipolia pensilis*. The larvae likely are general feeders and may ascend shrubs to feed. *Lacinipolia acutipennis* flies in late summer with most specimens recorded from mid-August to late September.

#### Remarks.

The name *acutipennis* has historically been associated with the taxon *Lacinipolia sareta* (i.e. *Lacinipolia vicina* of authors) rather than *Lacinipolia pensilis*. This apparently stemmed from the fact that historical *Lacinipolia acutipennis* specimens from western Nevada (the type locality of *Lacinipolia acutipennis*) and adjacent northeastern California had been wrongly associated; a series from Truckee, California, examined by Lloyd Martin (and probably others before him, including McDunnough) consists of male *Lacinipolia sareta* and female *Lacinipolia acutipennis*, but only the male *Lacinipolia sareta* were previously dissected. Female *Lacinipolia sareta* from the northern Sierra Nevada and especially Nevada are considerably paler. Comparison of the type female of *Lacinipolia acutipennis* to all other *Lacinipolia vicina*-group taxa occurring in the region of the type locality shows that *Lacinipolia acutipennis* is a dark female of the low-elevation taxon previously treated as a form of *Lacinipolia pensilis*.

Variation in the DNA barcodes (Fig. 75) could be indicative of cryptic species, but genitalic structure is highly conserved and phenotypic blending is apparent from regions where adequate samples were available.

### Lacinipolia sareta

Taxon classificationAnimaliaLepidopteraNoctuidae

(Smith, 1906)

Figs 7–15 , 59 , 67 , 73

Mamestra sareta Smith, 1906: 229.

#### Type material.

lectotype male (AMNH, examined), designated by Todd (1982); type locality: Minnehaha, Yavapai Co., Arizona.

#### Diagnosis.

*Lacinipolia sareta* is the most common and widespread species in the *Lacinipolia pensilis* group, and most of the identification difficulties are in separating it from *Lacinipolia pensilis* and *Lacinipolia acutipennis* in the West. This is most reliably done based on genitalia, where males lack the ventrally projecting, paired spinose crests above the juxta that are found in *Lacinipolia acutipennis* and *Lacinipolia pensilis*; females of *Lacinipolia sareta* have a simple ostium with a strongly convex prevaginal margin, compared to those of *Lacinipolia pensilis* and *Lacinipolia acutipennis* which have an asymmetrical, conch-shaped ostium with a straight prevaginal margin. *Lacinipolia sareta* flies earlier in the year (mostly June–July) than *Lacinipolia pensilis* and *Lacinipolia acutipennis* (August–September), although the southernmost *Lacinipolia sareta* populations in Arizona, New Mexico, and Texas fly again in late September–October after an initial May flight.

The remaining species (*Lacinipolia vicina*, *Lacinipolia teligera*, and *Lacinipolia dimocki*) can, for the most part, be distinguished from *Lacinipolia sareta* by geographic distribution; in Washington, Oregon and California, where the range of *Lacinipolia sareta* overlaps that of *Lacinipolia dimocki*, *Lacinipolia sareta* is smaller and has a duller white hindwing, in addition to the genitalic characters given under *dimocki*. From eastern Colorado and New Mexico through western Oklahoma and northern Texas *Lacinipolia sareta* overlaps with *Lacinipolia teligera*; characters given in the keys and the *Lacinipolia teligera* diagnosis will separate the two. The range of *Lacinipolia sareta* might overlap with that of *Lacinipolia vicina* in the East (from the Great Lakes region eastward through New York and New England), where the smaller size, different flight period and genitalic differences given under *Lacinipolia vicina* will reliably separate the two.

#### Distribution and biology.

*Lacinipolia sareta* occurs throughout western North America from the southern Yukon and Northwest Territories to Texas, Arizona and California; it undoubtedly also occurs in northern Mexico. It ranges eastward across the southern boreal region to at least Quebec, with an unverified record from Maine (Forbes 1954). Most or all records of *Lacinipolia vicina* for Michigan (Moore 1955) probably apply to this species, but *Lacinipolia sareta* is not known from Ohio (Rings et al. 1992) where it would be expected in sandy habitats along Lake Erie. Although found in a huge variety of woodland, steppe and prairie habitats, *Lacinipolia sareta* particularly favours sandy soils and can be abundant in dune and beach habitats. Crumb (1956) describes the ground-dwelling, polyphagous larva (as *Lacinipolia vicina*). Godfrey (1972) illustrates the larva, and states that Arizona and Montana larvae are identical.

#### Remarks.

The vast geographic range and considerable DNA barcode variation suggest that *Lacinipolia sareta* could be a cryptic species complex. Alternatively, DNA barcode variation simply may not be fully congruent with species limits in the group, a phenomenon that occurs in about 10% of Noctuoidea (Zahiri et al. 2014). In contrast to the mtDNA variation, genitalic structure and wing pattern is highly conserved, and I could find no way to segregate specimens with divergent barcodes or those from different ecoregions. The shape of the digitus varies somewhat, with nominate *Lacinipolia sareta* from the southwestern United States with a slightly longer, narrower tine-like digitus, compared to most (but not all) northern specimens, which have a shorter more triangular digitus, but the differences are inconsistent and again do not correlate with geographic or molecular differences. This is surprising given that molecular divergence among *Lacinipolia sareta* haplogroups was greater than the minimum divergence between *Lacinipolia sareta* and *Lacinipolia dimocki* (Fig. 75), despite the considerable morphological differences between the two species. Studies of pheromone variation, nuclear DNA sequence data, and immature stages would provide more insight into this difficult group.

### Lacinipolia dimocki

Taxon classificationAnimaliaLepidopteraNoctuidae

Schmidt sp. n.

http://zoobank.org/3818A542-7458-4999-9547-F2DCFB980F1B

Figs 16–21 , 60 , 68 , 74

#### Type material.

**Holotype** ♂. California: Ventura Co., Cuyama Valley, Apache Canyon, 0.6 mi E of Hwy. 33, 34.751193°N, 119.399772°W, 3497’, 19.Jun.2009, T. E. Dimock [CNC]. **Paratypes** 17♂ 18♀. Same data as holotype, 2♂ 7♀; California: Ventura Co., Pine Mountain, Pine Mountain campground, 6620’, 21.Aug.2000, T. E. Dimock, 1♂ 1♀, 27.Jun.2000, 2♀; Ventura Co., Sespe Creek at Derrydale Creek, 34.583992°N, 119.262757°W, 15.Jul.2009, T. E. Dimock, 2♂ 2♀; Ventura Co., Sespe Creek at Tule Creek, 34.561350°N, 119.264780°W, 1♂; Ventura Co., Upper Ojai Valley, 34.451°N, 119.121°W, 2120’, 31.May.2003, T. E. Dimock, 1♂; Ventura Co., Cuyama Valley, 34.695°N, 119.398°W, 3540’, 14.Jun.2005, T. E. Dimock, 3♂; San Diego Co., Laguna Mountains, Pine Creek Road, 5500’, 1.Jul.2000, T. Mustelin, 2♂; same data, DNA barcode vouchers # CNCNoctuoidea7969 and CNC LEP00053134, 2♂; same locality, 29.Aug.2000, 1♀; San Diego Co., Laguna Mountains, Desertview Overlook, 5800’, 29.Aug.2000, T. Mustelin, 1♂ 1♀; San Diego Co., Laguna Mountains, Kitchen Creek Road, 5500’, 29.Aug.2000, T. Mustelin, 1♂ 1♀; San Bernardino Co., San Bernardino Mountains, Cactus Flats, 34°18.32’ N 116°47.99’ W, 6100’, 25.May.2006, T. Mustelin, 1♀; San Bernardino Co., San Bernardino Mountains, Onyx Summit, 34°11.50’ N 116°43.06’ W, 8500’, 25.May.2006, T. Mustelin, 1♀; 1.Aug.2006, 1♀; Riverside Co., Pinyon Crest, 33.614N 116.446 W 4200’, 29.Sep.2001, R. Leuschner, 1♂. CNC, USNM. The type material is restricted to specimens from southern California.

#### Etymology.

This species is named in honour of Thomas E. Dimock for his contributions to the knowledge of southern California moths. His efforts to collect research specimens provided most of the type series of *Lacinipolia dimocki*.

#### Diagnosis.

This western species was previously included with *Lacinipolia sareta* (*Lacinipolia vicina* of authors), but it is a cryptic, mostly parapatric species that replaces *Lacinipolia sareta* from the Washington coast ranges southward through California. The two occur sympatrically in south-central Washington, and possibly elsewhere along the interface of the Great Basin–Coast Ranges and Sierra Nevada. Externally *Lacinipolia dimocki* is larger with an overall paler, less contrasting forewing pattern and usually a lighter, more pearly-white hindwing. The male genitalia differ in having a sinuate, tine-like clasper rather than the flattened, two-lobed clasper of *Lacinipolia sareta*; also the ventral swelling of the phallus is much more pronounced in *Lacinipolia dimocki*. Females can be difficult to separate from those of *Lacinipolia sareta*; in addition to the forewing characters mentioned above, *Lacinipolia dimocki* is generally larger overall and with a less sinuous, less dorsoventrally flattened ductus bursae and a relatively larger corpus bursae.

#### Description.

**Head.** Antenna of male appearing filiform, but slightly serrate under magnification; antenna of female filiform; dorsal scaling grey; scape, and vertex with a mix of dull-white and dark-grey scales, these spatulate and bifid apically; frons with thin white, strap-like scales, bordered by transverse band of dark-grey scales at dorsal margin; labial palpi with mix of dull-white and dark-grey scales; 3^rd^ segment 0.4× length of 2^nd^ segment. **Thorax.** Vestiture of light-grey scales tipped with dark-grey apex; tegula and patagium with subterminal border of black scales, border of the tegula diffuse, but that of patagium forming distinct black prothoracic line; caudal margin with slight tuft; legs with mix of light- and dark-grey scales, tarsi with slight banding pattern formed by border of lighter scales along distal margin of each tarsal segment. **Wings.** Average forewing length of males 15.0 mm (n = 9, range 14.2–15.8 mm), females 15.1 mm (n = 9, range 13.8–16.9 mm); forewing ground colour pale grey, medial area pale grey brown; antemedial and postmedial line incomplete or absent, when present then best developed toward anal margin and fading out towards costa, antemedial line double, sometimes with slightly paler grey infill; postmedial line double, often forming pale, indistinct crescent opposite claviform spot; subterminal area with diffuse dark shading in subapical and anal areas, latter sometimes with a small white crescent; basal dash black and crisp; orbicular spot slightly oblong to slightly kidney shaped, with incomplete, thin black border and interior slightly paler than ground colour; reniform spot with incomplete thin black border, interior slightly paler than ground, with indistinct, darker inner ring; claviform usually distinct, forming a thin black, open V; fringe dark grey, pale grey at vein terminus resulting in indistinct striping; male hindwing bright, slightly pearlescent white with terminal third of veins, and thin diffuse margin fuscous; female hindwing duller white overall with more extensive fuscous shading on veins and marginal area. **Abdomen.** Vestiture light grey, first four segments with slight dorsal tufts of darker grey scales; tuft of 4^th^ segment most prominent. **Male genitalia.** Uncus slender, 10–11× longer than wide, evenly tapered from base to apex, with sparse long setae directed basad; valve with extreme subapical constriction forming a narrow neck, such that apex consists of strongly spatulate cucullus; valve abruptly angled caudoventrally beyond apical third; cucullus anvil shaped, interior surface densely covered with fine long hairs; corona consisting of a single row of flattened marginal spines, and a cluster of spines in tip of caudoventral lobe; sacullus with membranous, rectangular flap (possibly a modified editum), which is densely covered in long setae; clasper forming a long, simple sinuate tine, extending to, or slightly beyond, costa; digitus a simple flattened lobe, 2× longer than wide; juxta with two lateral, rounded triangular plates flanking phallus, these with short, straight dorsally directed spines on inner surface; phallus with ventral swelling 2/3 from base; apical third curving ventrad slightly; phallus with small, broad-based, thorn-like dorsal cornutus at apical ¾; vesica directed left-ventrad, then coiling dorsad and forming extended spiral through one rotation; vesica with small medial patch of spinules, and larger preapical patch extending slightly along axis of vesica. **Female genitalia.** Bursa copulatrix unisaccate; ductus bursae moderately sclerotized and dorsoventrally flattened, 5× longer than wide; corpus bursae globose, membranous and slightly corrugated, lacking signa; appendix bursae slightly coiled, with ductus seminalis situated preapically; ostium bursae extending caudad as an invaginated slit; prevaginal margin convex and slightly rounded-conical; terminal segments telescopic, with posterior apophysis twice as long as anterior apophysis; papillae small, narrow and lobe-like, membranous and moderately setose.

#### Distribution and biology.

The early stages and larval food plants are unknown, but like other species in the group, larvae of *Lacinipolia dimocki* probably are ground-dwelling and polyphagous on herbaceous plants. It occurs from the east slope of the Washington Coast Ranges to southern California.

## Supplementary Material

XML Treatment for Lacinipolia vicina

XML Treatment for Lacinipolia teligera

XML Treatment for Lacinipolia pensilis

XML Treatment for Lacinipolia acutipennis

XML Treatment for Lacinipolia sareta

XML Treatment for Lacinipolia dimocki

## Footnotes

1 The spine field above the juxta and even the aedeagal cornuti can often be examined without dissection by carefully removing the terminal abdominal hairs with a small brush, especially if the valves have previously been spread by squeezing the base of the genital capsule when specimen is still fresh.

## Supplementary materials

Supplementary material 1 Table S1. Specimen data for mtDNA barcode vouchers Data type: data spreadsheet Explanation note: Haplogroup numbers refer to those given in Fig. 75. Abbreviations for specimen depositories are as given in Methods and materials section. This dataset is made available under the Open Database License (http://opendatacommons.org/licenses/odbl/1.0/). The Open Database License (ODbL) is a license agreement intended to allow users to freely share, modify, and use this Dataset while maintaining this same freedom for others, provided that the original source and author(s) are credited.B. Christian Schmidt
